# PDGF-BB accelerates TSCC via fibroblast lactates limiting miR-26a-5p and boosting mitophagy

**DOI:** 10.1186/s12935-023-03172-6

**Published:** 2024-01-02

**Authors:** Jianguo Xu, Li Bian, Dingyun You, Ziliang Li, Tingting Wang, Yiting Li, Xiaobin Ren, Yongwen He

**Affiliations:** 1 Department of Oral and Maxillofacial Surgery, Kunming Medical University School and Hospital of Stomatology, Kunming, 650106 China; 2 Yunnan Key Laboratory of Stomatology, Kunming, 650106 China; 3 Department of Pathology, The First Affiliated Hospital of Kunming Medical University, Kunming, 650106 China; 4 School of Public Health, Kunming Medical University, Kunming, 650500 China; 5 Department of oral Implantology, Kunming Medical University School and Hospital of Stomatology, Kunming, 650106 China; 6 Department of Stomatology, The First People’s Hospital of Yunnan Province, Kunming, 650032 China; 7 Department of Periodontology, Kunming Medical University School and Hospital of Stomatology, 1088 Haiyuan Central Road, Kunming, Yunnan 650106 China; 8 Department of Dental Research, Kunming Medical University School and Hospital of Stomatology, 1088 Haiyuan Central Road, Kunming, Yunnan 650106 China; 9 Qujing Medical College, Qujing, 655011 China

**Keywords:** PDGF-BB, Lactate, Mitophagy, Cancer-associated fibroblasts, Metabolism reprogramming

## Abstract

The tumor microenvironment and cancer-associated fibroblasts (CAFs) play crucial roles in tumor development, and their metabolic coupling remains unclear. Clinical data showed a positive correlation between PDGF-BB, CAFs, and glycolysis in the tumor microenvironment of oral tongue squamous cell carcinoma patients. In vitro, CAFs are derived from hOMF cells treated with PDGF-BB, which induces their formation and promotes aerobic glycolysis. Mitophagy increased the PDGF-BB-induced formation of CAF phenotypes and aerobic glycolysis, while autophagy inhibition blocked PDGF-BB-induced effects. Downregulation of miR-26a-5p was observed in CAFs; upregulation of miR-26a-5p inhibited the expression of mitophagy-related proteins ULKI, Parkin, PINK1, and LC3 and aerobic glycolysis in PDGF-BB-induced CAFs. PDGF-BB-induced CAFs promoted tumor cell proliferation, invasion, metastasis, NF-κB signaling pathway activation, and PDGF-BB secretion. Thus, PDGF-BB is associated with lactate-induced CAF formation and glucose metabolism reprogramming. These findings indicate potential therapeutic targets in oral tongue squamous cell carcinoma.

**Supplementary Information:**

The online version contains supplementary material available at 10.1186/s12935-023-03172-6.

## Introduction

The tumor microenvironment is the internal environment of a tumor during its development, comprising various cell types, such as tumor cells, stromal cells, vascular endothelial cells, immune cells, and extracellular matrix. The mode of communication between these cells depends on the metabolic activity of the tumor. Hypoxia, an acidic extracellular environment, and nutrient deficiencies are major stress factors characterizing the tumor microenvironment [1]. The metabolic pattern of cancer-associated fibroblasts (CAFs) has also been found to be altered, and their crosstalk with cancer cells can significantly impact the occurrence, development, metastasis, and treatment of tumors. Thus, understanding the underlying mechanisms associated with CAFs has become a major focus of research in the field of tumor biology [2].

CAFs can originate from various cell types, such as host fibroblasts, mesenchymal stem cells, epithelial cells, endothelial cells, and perivascular cells [3]. The main source of CAFs is host fibroblasts that have undergone transformation induced by factors secreted by tumor cells. Among these factors, platelet-derived growth factor-BB (PDGF-BB) is a crucial cytokine closely linked to tumorigenesis and development. When PDGF-BB binds to its receptor, it initiates and amplifies signals that promote growth, induce actin rearrangement, and play a chemotactic role through the specific phosphorylation of tyrosine residues. Research has demonstrated that PDGF-BB can induce the proliferation and migration of vascular endothelial cells, smooth cells, and tumor cells, while also inhibiting their apoptosis [4]. In addition, PDGF-BB can recruit pericytes, endothelial cells, and fibroblasts to the tumor site to form the tumor stroma [5, 6].

Glucose is a crucial carbon source and energy substance for cells that generates energy through glycolysis and oxidative phosphorylation in mitochondria and synthesizes biological macromolecules and reduction equivalents through the pentose phosphate pathway, supporting cell growth and proliferation [7]. Glycolysis has been thought to be preferred by tumor cells for energy production to meet their rapid proliferation needs, even under aerobic conditions, and the role of oxidative phosphorylation is considered low. This phenomenon is known as the “Warburg effect” [8]. Recent studies have revealed that activated fibroblasts also undergo glucose metabolism reprogramming, switching from oxidative phosphorylation to aerobic glycolysis, similar to the “Warburg effect” of tumor cells. This process produces lactic acid and high-energy metabolites, such as ketones, which can be transferred to adjacent tumor cells and enter the tricarboxylic acid (TCA) cycle, increasing oxidative phosphorylation and ATP production. This process enables tumor cells to switch from aerobic glycolysis to oxidative phosphorylation to acquire energy, resulting in the “reverse Warburg effect” [9, 10]. Notably, both tumor cells and CAFs tend to exhibit aerobic glycolysis. However, the proliferation rate of CAFs is surprisingly lower than that of normal fibroblasts, which contrasts with the rapid proliferation of cancer cells. This difference indicates that aerobic glycolysis plays different roles in CAFs and tumor cells, and the carbohydrates produced by aerobic glycolysis are not used in the biosynthesis of CAFs [11].

Mitochondria are important energy-supplying organelles in eukaryotes and are critical for processes such as intracellular energy production, metabolism, and cell death. Mitochondrial autophagy is an important regulator of mitochondrial homeostasis [12]. Mitochondrial autophagy leads to disorders of pyruvate metabolism, increasing glucose consumption and lactic acid production, reducing oxidative respiration capacity and ATP production, inhibiting cellular oxidative phosphorylation inhibition, and enhancing glycolysis [13]. Studies have shown that mitochondrial autophagy is closely related to tumorigenesis and treatment effects; autophagy exists not only in tumor cells but also in stromal cells in the tumor microenvironment [14]. Cancer cells can induce mitochondrial autophagy in nearby CAFs, reducing intracellular mitochondria and increasing the expression level of enolase-1 and fructose diphosphate aldolization glycolytic enzyme [15]. Autophagy of CAFs can affect the secretion of cytokines, promoting tumor invasion and metastasis, and downregulating autophagy in CAFs has been found to inhibit tumor development [16].

Mitochondrial autophagy blocks oxidative phosphorylation, enhances aerobic glycolysis, and leads to the secretion of large concentrations of lactic acid; thus, it is an important component of tumor microenvironment stress. In the tumor microenvironment, cancer-associated fibroblasts (CAFs) secrete high levels of lactic acid through glycolysis. While the lactic acid produced by glycolysis is primarily exported via MCT-4, the lactic acid outside of cancer cells is transported into these cells through MCT-1. Once inside, lactate is converted into pyruvate by lactate dehydrogenase B (LDHB) and subsequently enters the mitochondrial tricarboxylic acid (TCA) cycle [17]; on the other hand, as a signaling molecule, it can promote tumor cell proliferation and migration, induce tumor cell epithelial–mesenchymal transition and dryness acquisition, promote tumor immune escape, and mediate chemoradiotherapy tolerance [18–22]. Tisato V demonstrated that NF-κB signaling activation can promote the transcription and expression of PDGF-BB [23]. The above results suggest that PDGF-BB induces activated fibroblasts to reprogram glucose metabolism to secrete lactate to feed cancer cells, further promoting the secretion of PDGF-BB by cancer cells and forming a positive feedback interaction loop to promote tumorigenesis and development. Therefore, whether this specific internal mechanism is related to the activation of the NF-κB signaling pathway is worth further study.

In summary, there is a symbiotic glucose metabolism–related interaction between tumor cellsand CAFs that promotes tumorigenesis and development; however, the underlying mechanism remains unknown. In the context of a multi-factorial tumor microenvironment, We hypothesize that PDGF-BB secreted by oral squamous cell carcinoma cells may induce fibroblast mitochondrial autophagy and switch glucose metabolism from oxidative phosphorylation to aerobic glycolysis, leading to the production of lactic acid. This lactic acid feeds back to cancer cells, forming a positive interaction feedback loop that promotes the occurrence, development, and metastasis of tumors. We recognize that this represents a simplified view of a complex system, and further research is needed to fully understand the intricacies of these interactions. To explore these scientific assumptions, we first measured the expression levels of relevant factors in clinical oral squamous cell carcinoma tissue specimens. We then established a PDGF-BB-induced fibroblast activation model to explore the metabolic coupling interactions and related signal transduction between PDGF-BB and lactic acid. We also established a nude mouse cotransplantation model to explore the mutual regulation function of oral squamous cell carcinoma and fibroblast metabolism in vivo. Our findings reveal that PDGF-BB and lactic acid mediate the symbiotic effect of glucose metabolism in oral squamous cell carcinoma cells and cancer-associated fibroblasts. This finding clarifies the mechanism of tumor progression from the perspective of metabolic interactions and their intrinsic relationship. These molecular events lay the foundation for exploring the reversal of tumorigenesis and development by targeting PDGF-BB and/or lactic acid.

## Materials and methods

### Collection of clinical samples

This study was approved by the Ethics Committee of Kunming Medical University, and written informed consent was obtained from all patients before the research started. The study was conducted in accordance with the recognized ethical principles described in the Declaration of Helsinki. Collectively, 43, 21, and 20 paraffin-embedded specimens diagnosed as oral squamous cell carcinoma were obtained at the Department of Pathology at the First Affiliated Hospital of Kunming Medical University, the Affiliated Stomatology Hospital of Kunming Medical University, and the West China Stomatology Hospital, respectively. Histologic and pathologic diagnoses were independently confirmed by at least two experienced histopathologists according to the sixth edition TNM classification criteria.

### Isolation of human normal primary fibroblasts and CAFs

CAFs and adjacent normal fibroblasts from oral tongue squamous cell carcinoma tissue were isolated in high-glucose Dulbecco’s Modified Eagle Medium (DMEM) supplemented with 10% FBS and 3% collagenase type II. After digestion, the tissue was cultured in a 10% bovine serum culture solution for 2–4 h at 37 °C. The fibroblasts were identified using immunofluorescent staining and cryopreserved in liquid nitrogen after three passages.

### Tumor xenograft model

All animals were housed in accordance with institutional guidelines approved by the Kunming Medical University Animal Care and Use Committee. Six-week-old male BALB/c nude mice were purchased and housed in a specific pathogen-free (SPF) animal laboratory. Four nude mice were randomly divided into four groups and subcutaneously injected in the left flank with a combination of hOMF/CAFs (5 × 10^6 cells) and Cal-27 (5 × 10^6 cells). Tumor volume was measured every 3 days, and the final tumor weight was recorded after sacrifice. All mice were sacrificed 24 days after injection, and the tumors were harvested and weighed. Tumor volume was calculated as V = L×W×W/2.

### Cell culture and treatment

The Human Oral Mucosa fibroblast cells (hOMF) and Oral Tongue Squamous Cell Carcinoma Cells(Cal-27) were purchased from the National Collection of Authenticated Cell Culture. The Normal Human Oral Mucosa (P2) 500 K Keratinocyte cells (hOMK) and Normal Human Oral Mucosa (P3) 500 K Fibroblast cells (hOMF) were obtained from www.Cellresearchcorp.com, while the Cal-27 cells from BioVector NTCC Inc. (Beijing, China).All cells were conventionally cultured in DMEM (Gibco, Grand Island, NY) supplemented with 10% heat-inactivated fetal bovine serum (Gibco), 2 mM L-glutamine (Gibco), 100 U/mL penicillin, and 100 µg/mL streptomycin (Gibco) at 37 °C with 5% CO_2_. CAFs were obtained by using recombinant human PDGF-BB for 72 h to activate conventionally cultured hOMFs and subsequently collected and analyzed the cells. Cells were cultured in serum-free DMEM containing 20 ng/ml PDGF-BB for 72 h to induce autophagy, as previously described [24]. To generate a conditioned medium from CAFs (CAF-CM), we mixed 2/3-volume CAF-supplemented medium with 1/3-volume DMEM low-glucose serum-free medium. To simulate a nutrient-deficient environment for tumor cells, use DMEM low-glucose medium serum-free (Starvation, Star) for a culture duration of 48 h.

### Cell proliferation assay

Cell proliferation was determined using Cell Counting Kit-8 (CCK-8 Kit, Beyotime Inst Biotech, China) according to the manufacturer’s instructions.

### Reagents

PDGF Receptor beta (ab32570), alpha smooth muscle Actin (ab5694), PDGF-BB (ab23914), α-SMA (ab5694), FAP-α (ab53066), β-Tubulin (ab179513), GLUT1 (ab115730), Bcl-2 (ab32124), DAPI, LDH-B (ab240482), LC3β (ab53066), Glut1 (ab115730), obtained from Abcam. Human Cytokine Antibody Array 1000 Chip (QAH-CAA-1000) purchased from Raybiotech, IgG (A7028) from R&D, Recombinant human PDGF-BB protein from Peprotech. Dylight 488 goat anti-mouse IgG, Dylight 649 goat anti-rabbit IgG from Abbkine. MCT4 (bs-2698R), LDHA (bs-18205R), Hexokinase II (bs-9455R), MCT1/SLC16A3 (bs-10249R) antibodies from Bioss. NF-κB p65 (GB11142), MT-ND1 pAb (GB113284), NF-κB p65 (phospho S536) antibodies from Servicebio. PINK1, Parkin, ULK1, LC3A/B, AMPK, from CST. CCCP, Mdivi-1, SC74751, α-cyano-4-hydroxycinnamate (CHC) from Selleck. Finally, Monodansylcadaverine (MDC) and Lactate from Sigma.

### Western blotting

Cells were lysed in RIPA cell lysis buffer (Kangwei, Beijing, China) containing a phosphatase inhibitor cocktail (Sigma). Equal protein concentrations were loaded onto 10% SDS-PAGE gels and then transferred onto 0.22 mm PVDF membranes (Millipore, MA, USA). The membranes were incubated with primary antibodies overnight at 4 °C and subsequently with HRP-conjugated secondary antibodies before visualization using ECL reagent (Millipore, MA, USA).

### Quantitative real-time polymerase chain reaction (qRT-PCR)

Total RNA was extracted using TRIzol reagent (Invitrogen, Carlsbad, CA) and reverse transcribed to cDNA using a Reverse Transcription system (Promega, Madison, WI) according to the manufacturer’s instructions. qRT-PCR was conducted using the SYBR Green Reagent kit (Applied Biosystems, Foster City, CA).

**Table:** 

Gene		Sequence(5ʹ→3ʹ)
has-miR-26a-5p	Forward primer	GGTTCAAGTAATCCAGGATAGGCT
has-miR-26a-5p	Reverse primer	TAGCACAGCCTGGATAGCAAC
U6	Forward primer	CTCGCTTCGGCAGCACAT
U6	Reverse primer	AACGCTTCACGAATTTGCGT
PDGF-BB	Forward primer	GCTTGGCTCGTGGAAGAAGGAG
PDGF-BB	Reverse primer	GGCGTTGGTGCGGTCTATGAG
GAPDH	Forward primer	AGGTCGGTGTGAACGGATTTG
GAPDH	Reverse primer	GGGGTCGTTGATGGCAACA
α-SMA	Forward primer	AGCGTGGCTATTCCTTCGTT
α-SMA	Reverse primer	TGAAGGATGGCTGGAACAGG
FAP	Forward primer	TTATGCTGGTCGCCTGTTGG
FAP	Reverse primer	AGGAGACCACCAGAGAGCATA

### Luciferase assay

Luciferase activity was measured using a Dual Luciferase Assay Kit (Promega, America) according to the manufacturer’s instructions. (The putative binding site was from 5ʹ-TACTTGA-3ʹ to 5ʹ-CTATAGT-3ʹ.) All experimental operations were performed by GenePharma (Shanghai, China).

### Plasmid construction and lentivirus Infection

Human miR-26a-5p and human MCT1 were inserted into the lentiviral expression vectors PLVX-EGFP-tagged 3 × Flag-PGK-Puro and pWPT-eGFP, respectively. Lentiviruses and retroviruses were generated using a polyethylenimine-based DNA transfection system according to the manufacturer’s instructions. hOMF and Cal-27 cells were transfected, and cells expressing the vectors were selected by puromycin treatment (Gibco) for 2–4 weeks. All experiments were conducted by GenePharma (Shanghai, China).

### Immune staining

was used for paraffin-embedded samples. After deparaffinization and rehydration, the tissue sections were incubated with antibodies overnight at 4 C, followed by incubation with peroxidase-labeled antibodies. Finally, antibody binding was visualized with a DAB substrate (ImmunoPure).

### Aerobic glycolysis analysis

Extracellular lactate was measured with a lactate assay kit (BioVision Technologies) according to the manufacturer’s instructions. The glucose uptake rate was determined as previously described (Yamamoto et al., 2011) using a specific kit (Glucose Uptake Colorimetric Assay Kit (Sigma Chemical MAK083)) according to the manufacturer’s instructions.

### Oxidative phosphorylation

Mitochondrial respiratory chain complexes I and II were measured using specific kits (mitochondrial respiratory chain complex I activity kit and mitochondrial respiratory chain complex II activity kit, NanJing JianCheng Bioengineering Institute NanJing, China) following the manufacturer’s instructions.

### Assessment of autophagy

For mitochondrial JC-1 fluorescent staining, cells were inoculated at 3 × 10^4^/mL, grown to 60% confluence, stained with 100 µL JC-1 working solution, washed twice with 1× Incubation Buffer, and observed under a fluorescent microscope. For MDC staining, cells were incubated with 100 µM MDC for 30 min in the dark at 37 °C, washed, and immediately analyzed under a fluorescence microscope.

### Transmission electron microscopy (TEM) for the observation of autophagic ultrastructure

The cells were fixed in EM fixative and centrifuged at low speed. The cells were then wrapped with agarose, rinsed with PB, fixed with 1% acetic acid 0.1 M PB, washed with 0.1 M PB, dehydrated, embedded, and polymerized at 60 °C for 48 h. The slices were stained with uranium and lead, dried overnight, and observed and analyzed by TEM.

### LC3A/B detection

Cells were fixed with 100% methanol and permeabilized with 0.5% Triton X-100 in PBS. After blocking with 10% sheep serum in PBS for 30 min, the cells were incubated overnight at 4 °C with LC3A/B antibody (1:100). Nuclei were stained with DAPI for 5 min in the dark, and images were acquired under a fluorescence microscope.

### Quantification of ATP, NADH, and PDGF-BB

ATP and NADH were measured using specific kits (ATP kit: Beyotime S0026, NADH kit: Beyotime S0175) according to the manufacturer’s instructions. PDGF-BB levels in supernatants were measured using a PDGF-BB enzyme-linked immunosorbent assay (ELISA) kit (RD Systems, DBB00) according to the manufacturer’s instructions.

### Wound healing assay

Cal-27 cells were cultured in six-well plates with a complete medium at a density of 4 × 10^5^ cells per well and incubated to 100% confluency. Then, we scraped the cells with a 200 µL tip and washed the wells three times with PBS. Finally, Cal-27 cells were cultured with CM and lactic acid for 24 h, and cell migration was assessed at 0, 6, 12, and 24 h. Cell migration was recorded with a microscope under four random fields of view.

### Transwell invasion assay

Cal-27 cells were resuspended in 200 µL conditioned medium (6 × 10^4^ cells/well) and placed in a Matrigel-lined Transwell upper chamber. Five hundred microliters of cell culture medium containing 10% FBS was added to the lower chamber. After 24 h of incubation, permeabilized Cal-27 cells were fixed with methanol and stained with 0.05% crystal violet. The cells that migrated through the membranes were counted and captured under a microscope in four random visual fields.

### Assessment of co-localization of mitophagy dye and lysosomes

The mitophagy phenomenon was confirmed using a fluorescence microscope by assessing the colocalization of Mitophagy Dye and lysosomes. Mitophagy was measured using specific kits (Mitophagy Detection Kit: DOJINDO MD01) according to the manufacturer’s instructions.

### Human cytokine antibody array 1000 chip (QAH-CAA-1000, Raybiotech) detection

One hundred microliters of sample diluent was added to each chip well and removed; 100 µl of sample was then added to each well, and the slide was incubated overnight. The slide was then incubated with Thermo Scientific Wellwash Versa. Seventy microliters of biotin-labeled antibody was added, and the slide was incubated at room temperature with shaking and washed, and 80 µl of 1500-fold diluted fluorescent agent-streptavidin was added to each well. The slide was incubated at room temperature for 1–2 h in the dark, washed, and assessed for fluorescence using the laser scanner Axon GenePix.

### Statistical analysis

All statistical analyses were performed using Prism 8.0 (GraphPad Software Inc.). Data are shown as the mean ± SD. The significance of intergroup differences was estimated with Student’s *t* test or one-way ANOVA. All results were reproduced across triplicate experiments. *P* < 0.05 was considered to indicate significance.

### Ethics approval and consent to participate

The current study was approved by the Ethics Committee of Kunming Medical University.( Approval Date: November 10, 2020 ;Acceptance Number: 2,020,159; Approval Document Number: kmmu2020507).

## Results

### Tongue squamous carcinoma cells promote the conversion of fibroblasts to CAFs through the high secretion of PDGF-BB

**Fig. 1:**
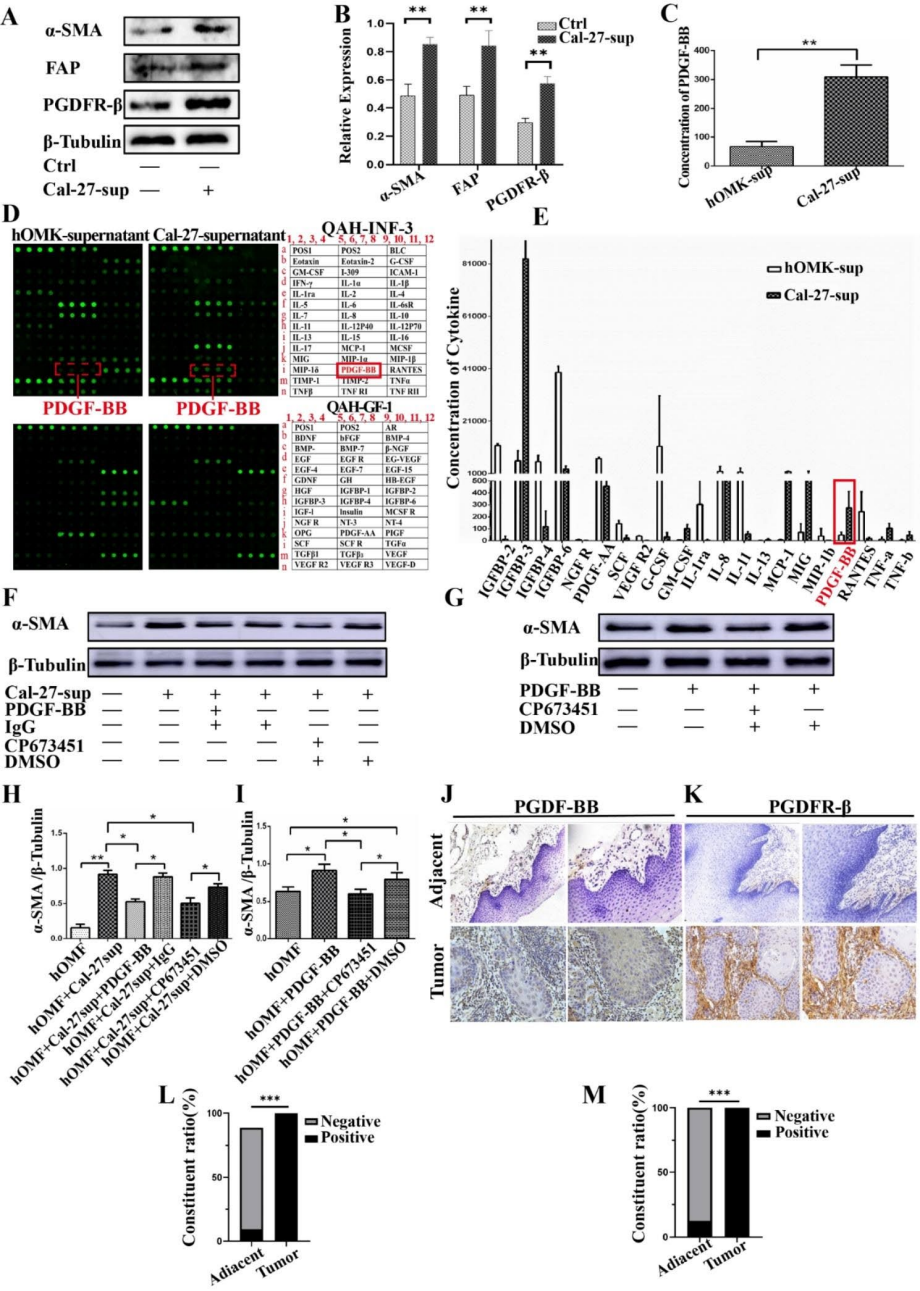
Western blotting revealed that Cal-27 supernatant induced elevated expression of fibroblast activation proteins α-SMA, FAP, and PDGFR-β (**A**, **B**). Protein microarray analysis demonstrated a significant 5.7-fold increase in PDGF-BB in this supernatant compared with that in the hOMK supernatant (**D**, **E**). This increase was further verified by ELISA (**C**). Western blotting also demonstrated that the use of a PDGF-BB antibody blocker and PDGFR-β receptor inhibitor attenuated the expression of fibroblast activation protein α-SMA induced by Cal-27 supernatant from tongue squamous carcinoma cells (**F**, **H**). Conversely, PDGF-BB induced fibroblast activation with high expression of α-SMA (**G**, **I**). In clinical specimens of tongue squamous carcinoma, PDGF-BB was found to be highly expressed in tumor cells (**J**, **L**), while PDGF-BR was mainly expressed in the tumor mesenchyme (**K**, **M**) (n = 24 adjacent tissue samples, 84 cancer tissue samples; magnification, 200× with a scale bar of 100 μm and magnification, 400× with a scale bar of 50 μm, respectively). The statistical analyses indicated significant differences (*P < 0.05, **P < 0.01, ***P < 0.001)

Previous research has demonstrated that tumor cells can activate fibroblasts to convert into CAFs by releasing various cytokines [25]. Here, we observed that the culture supernatant derived from tongue squamous cell carcinoma Cal-27 cells could induce the activation of fibroblasts and lead to high expression of α-SMA, FAP, and PDGFR-β (Fig. 1A-B). To further investigate the components responsible for this activation, we analyzed the supernatant using a protein microarray. Our results showed a significant 5.7-fold increase in PDGF-BB in the supernatant from Cal-27 cells versus that from oral mucosal epithelial cells (hOMK) (Fig. 1D and E). These findings were further verified using the ELISA method (Fig. 1C). To confirm the role of PDGF-BB in the activation of fibroblasts, we used a PDGF-BB antibody blocker and PDGFR-β receptor inhibitor (CP673451), which attenuated the expression of α-SMA in the supernatant of tongue squamous cell carcinoma Cal-27-induced fibroblasts (Fig. 1F, H). In addition, we observed that PDGF-BB alone could induce fibroblast activation and increase α-SMA expression (Fig. 1G, I). We then analyzed clinical samples of tongue squamous cell carcinoma and found high expression of PDGF-BB in tumor cells (Fig. 1J, L), while PDGF-BR was mainly expressed in the tumor stroma (Fig. 1K, M). These results demonstrate that tongue squamous cell carcinoma cells induce the transformation of fibroblast CAFs by an autocrine secretion of high levels of the cytokine PDGF-BB.

### PDGF-BB induces glycolytic reprogramming in activated fibroblasts

**Fig. 2:**
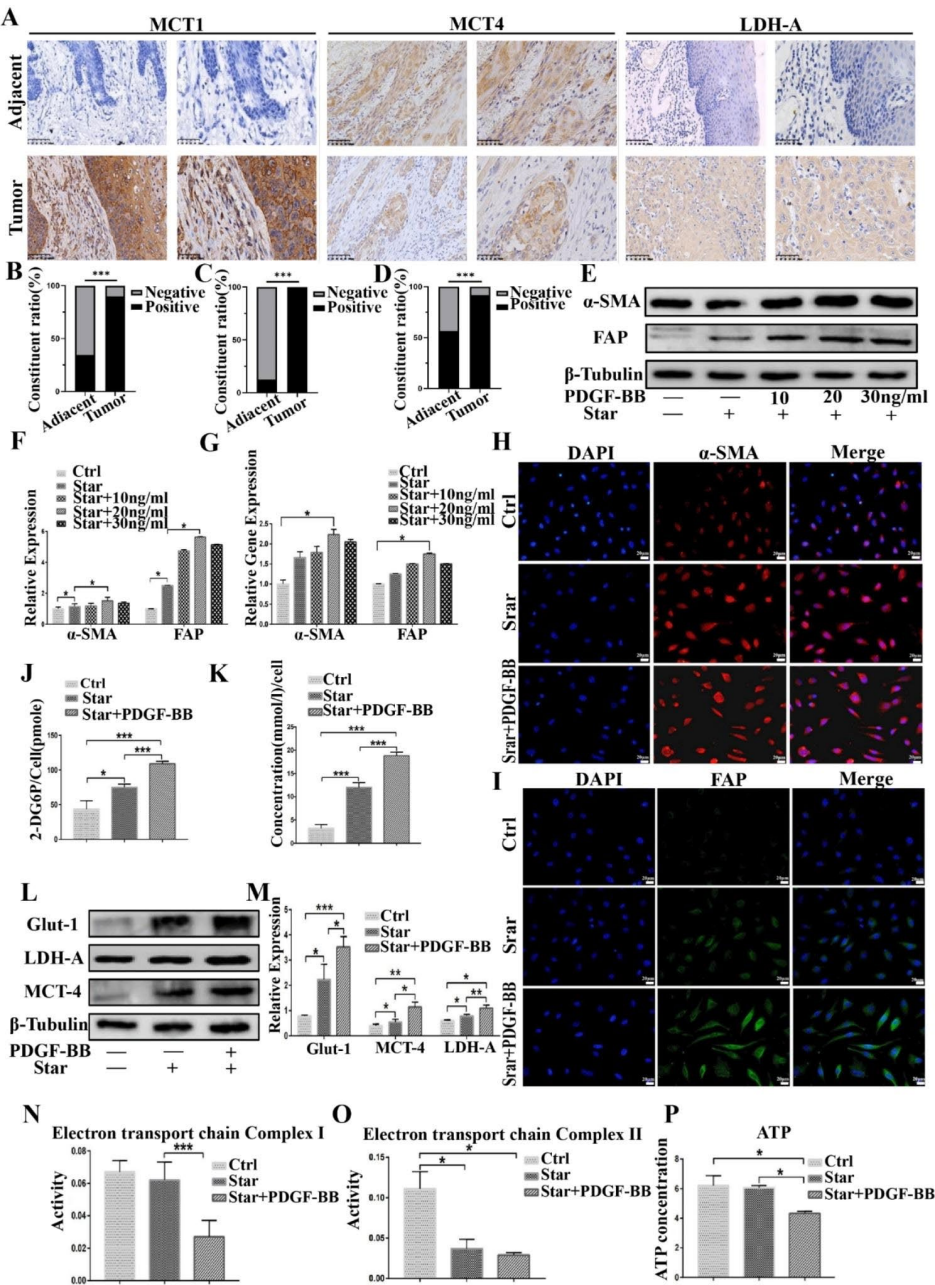
Tissue samples from oral squamous cell carcinoma demonstrated significantly higher expression levels of MCT1, MCT4, and LDHA in tumor tissues compared with those in paraneoplastic tissues (n = 32 adjacent tissue samples, 40 cancer tissue samples; magnification, 200× with a scale bar of 100 μm and magnification, 400× with a scale bar of 50 μm, respectively) (**A**–**D**). Western blotting showed that PDGF-BB induced high α-SMA and FAP expression levels in hOMF cells (**E**, **F**). qRT-PCR also demonstrated that PDGF-BB induced high α-SMA and FAP expression levels in hOMF cells (**G**). Further confirmation of the characteristics of CAFs was obtained by immunofluorescence microscopy (scale bar, 20 μm) (**H**, **I**). PDGF-BB significantly promoted glucose uptake (**J**) and lactate secretion (**K**) in starved cells. Western blotting demonstrated that PDGF-BB promoted the expression of GLUT-1, LDH-A, and MCT-1 (**L**, **M**). In addition, PDGF-BB inhibited the activity of mitochondrial respiratory chain complex I (**N**) and mitochondrial respiratory chain complex II (**O**) and decreased intracellular ATP content (**P**)

The Warburg and anti-Warburg effects are important forms of energy interaction between tumor cells and stromal cells in response to nutritional deficiencies in the tumor microenvironment [26]. We investigated the expression of MCT1, MCT4, and LDH-A in oral squamous cell carcinoma tissue samples and found that their expression in tumor tissues was significantly higher than that in paracancerous tissues, with expression observed in both the cytoplasm and membrane of cancer and stromal cells. MCT1 expression was significantly enhanced in cancer cells at the infiltration front, while MCT4 expression was stronger in cancer cells at the center of the cancer nest (Fig. 2A–D). We used hOMF cells challenged with starvation as an in vitro model to simulate the nutritional deprivation of the tumor microenvironment. The starvation condition was achieved using serum-free medium (Starvation, Star). Western blotting and qRT-PCR showed that PDGF-BB induced a CAF phenotype in starved hOMF cells, characterized by positive expression of α-SMA and FAP (Fig. 2E–G). These CAF features were further confirmed by immunofluorescent microscopy (Fig. 2H–I).

Previous studies have shown that tumor cells and stromal cells, especially fibroblasts, undergo metabolic reprogramming (such as glucose metabolism alterations) in response to nutritional deficiencies in the microenvironment. Therefore, we tested glucose metabolism-related indicators. Our results showed that PDGF-BB significantly promoted glucose intake (Fig. 2J) and lactic acid secretion in starved cells (Fig. 2K). Western blotting confirmed that PDGF-BB promoted the expression of glucose transporter protein-1 (GLUT-1), lactate production key enzyme lactate dehydrogenase A (LDH-A), and lactate conversion protein MCT-1 (Fig. 2L and M). To further demonstrate that PDGF-BB induces this metabolic transformation, we examined the activity of aerobic phosphorylation-related indicators of mitochondrial respiratory chain complex I and II and intracellular ATP content. The results showed that PDGF-BB inhibited the activity of mitochondrial respiratory chain complex I (Fig. 2N) and II (Fig. 2O) and reduced intracellular ATP content (Fig. 2P). Our results indicate that PDGF-BB can not only promote the activation of fibroblasts but also promote the conversion of glucose metabolism from aerobic phosphorylation to aerobic glycolysis.

### PDGF-BB enhanced mitophagy in Star-treated hOMFs

**Fig. 3:**
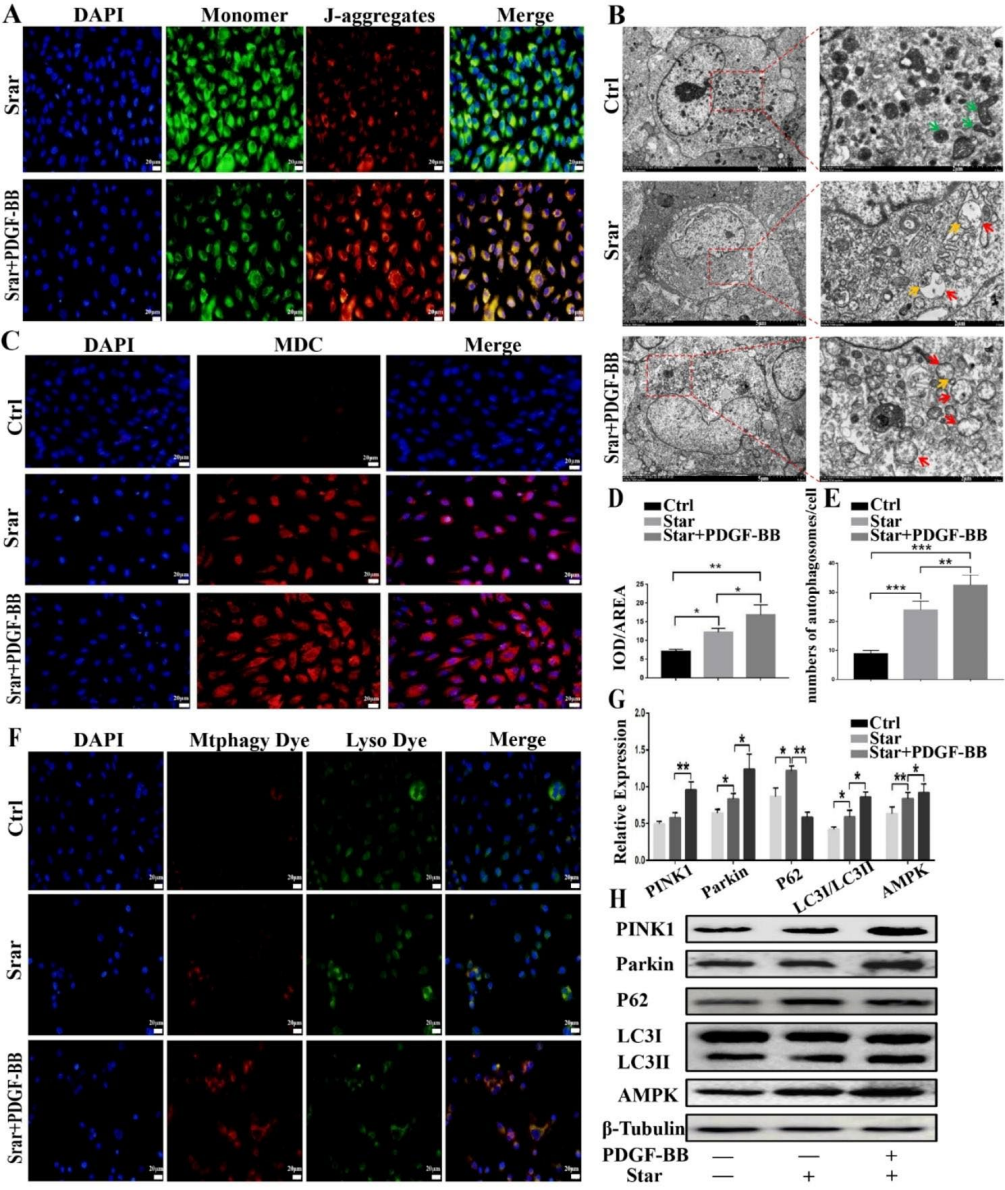
JC-1 staining revealed that serum-free incubation of hOMFs decreased the mitochondrial membrane potential (increased green fluorescence and decreased red fluorescence), while PDGF-BB had a protective effect (**A**). PDGF-BB also increased the proportion of MDC staining in cells and promoted autophagy (**C**, **D**). Transmission electron microscopy (TEM) revealed that control cells exhibited a normal tubular mitochondrial network (indicated by yellow arrows), whereas in Star-treated cells, deformed mitochondria were taken up by autophagosomes (indicated by red arrows). Furthermore, PDGF-BB increased the number of autophagic vesicles containing damaged mitochondria and repaired damaged mitochondria in Star-treated hOMFs (**B**). Fluorescence co-localization demonstrated that PDGF-BB promoted the binding of autophagic mitochondria and lysosomes (**E**, **F**). Western blotting showed that the treatment of hOMFs with Star promoted the protein expression of LC3-II, whereas PDGF-BB treatment significantly enhanced the protein expression of LC3-II, PINK1, and Parkin, and decreased the protein expression of LC3-I and P62 (**G**, **H**)

Mitochondria are essential for providing energy to eukaryotic cells and play a critical role in intracellular energy production. Maintaining mitochondrial homeostasis is important, and mitochondrial autophagy is one way to achieve this. Six different methods were employed to evaluate the level of mitophagy to determine whether PDGF-BB induces mitophagy in Star-treated hOMF cells. First, JC-1 staining showed that serum-free incubation of hOMF cells for 72 h reduced the mitochondrial membrane potential (green fluorescence increased and red fluorescence decreased), but PDGF-BB (20 ng/ml) had a protective effect (Fig. 3A). In addition, starvation promoted autophagy, as evidenced by the increased MDC positivity ratio. The presence of PDGF-BB increased the ratio of MDC staining in Star-stressed cells (Fig. 3C, D).

Next, microtubule-associated protein LC3A/B and transmission electron microscopy were used to detect autophagy, and the results confirmed the autophagy-promoting role of PDGF-BB. TEM showed that control cells exhibited a normal tubular mitochondrial network (yellow arrows), whereas Star-treated cells packaged deformed mitochondria in autophagosomes (red arrows). PDGF-BB increased the autophagic vacuole containing injured mitochondria and repaired them in Star-treated hOMFs (Fig. 3B). Third, the colocalization of mitochondrial autophagy and lysosomes was examined, and the results showed that PDGF-BB promoted the binding of autophagic mitochondria and lysosomes (Fig. 3E, F).

Finally, the occurrence of mitophagy was confirmed by Western blotting. Treatment of hOMFs with Star resulted in a slight accumulation of LC3-II. However, PDGF-BB treatment significantly enhanced LC3-II accumulation, with a concomitant decrease in LC3-I. The protein expression levels of PINK1 and Parkin were also increased, while P62 levels were reduced by PDGF-BB treatment (Fig. 3G, H).

### Mitophagy-mediated PDGF-BB-induced activation and aerobic glycolysis in Star-treated hOMFs

**Fig. 4:**
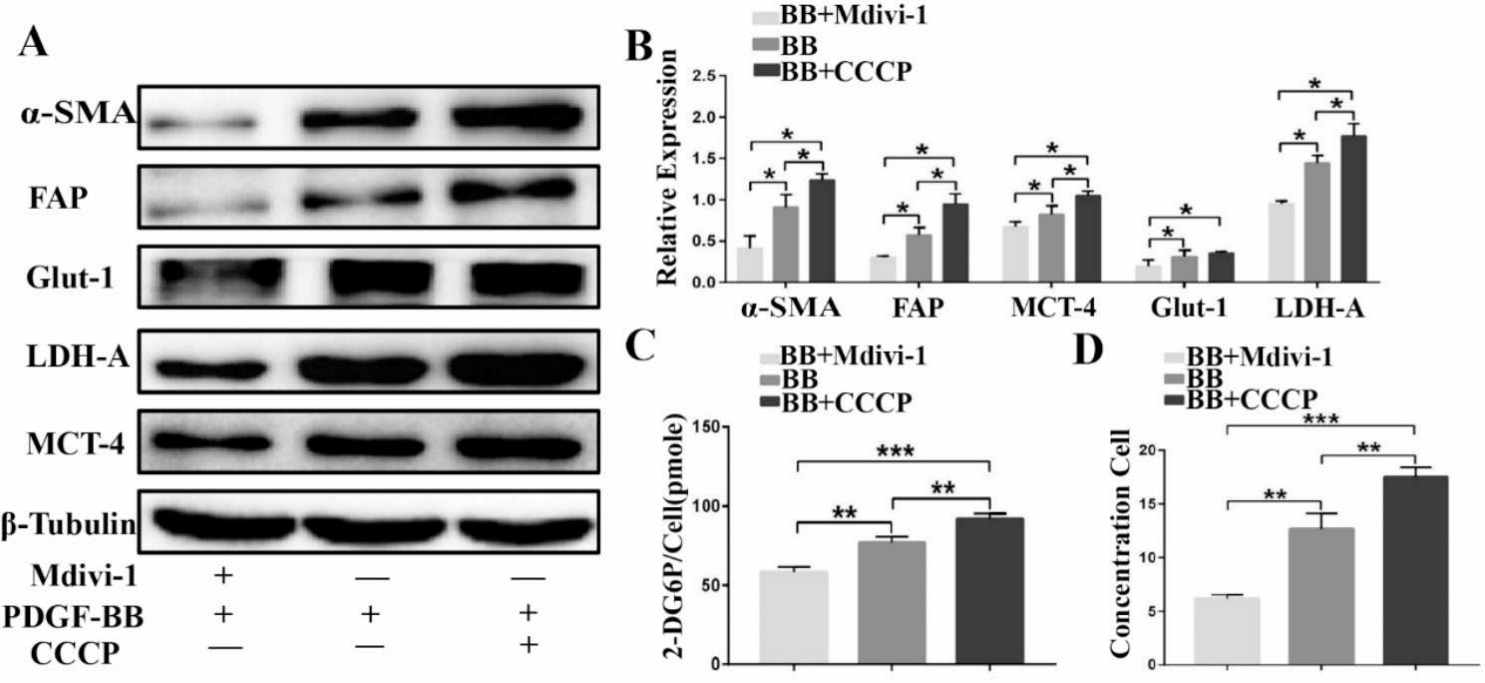
Western blotting revealed that treatment with mdivi-1, a mitochondrial autophagy inhibitor, and CCCP, an enhancer, significantly downregulated the protein expression of α-SMA, FAP, Glut-1, MCT-4, and LDH-A (**A**, **B**). In addition, mdivi-1 treatment led to decreased glucose uptake and lactate secretion in fibroblasts (**C**, **D**)

To provide a clearer explanation of the role of autophagy in PDGF-BB-induced activation and glucose metabolism, we utilized two compounds—the mitophagy inhibitor mdivi-1 and the enhancer CCCP. Our results demonstrated that mdivi-1 caused a significant downregulation of the expression of CAF markers α-SMA and FAP and inhibited the expression of Glut-1, MCT-4, and LDH-A (as illustrated in Fig. 4A, B). Moreover, the application of mdivi-1 also led to a decrease in glucose uptake and secretion of lactic acid, as indicated in Fig. 4C, D. These findings suggest that autophagy plays a crucial role in PDGF-BB-induced activation and glucose metabolism, and the inhibition of mitophagy may serve as a potential therapeutic approach for the treatment of PDGF-BB-induced diseases.

### PDGF-BB promotes mitophagy and aerobic glycolysis in primary cultured CAFs

**Fig. 5:**
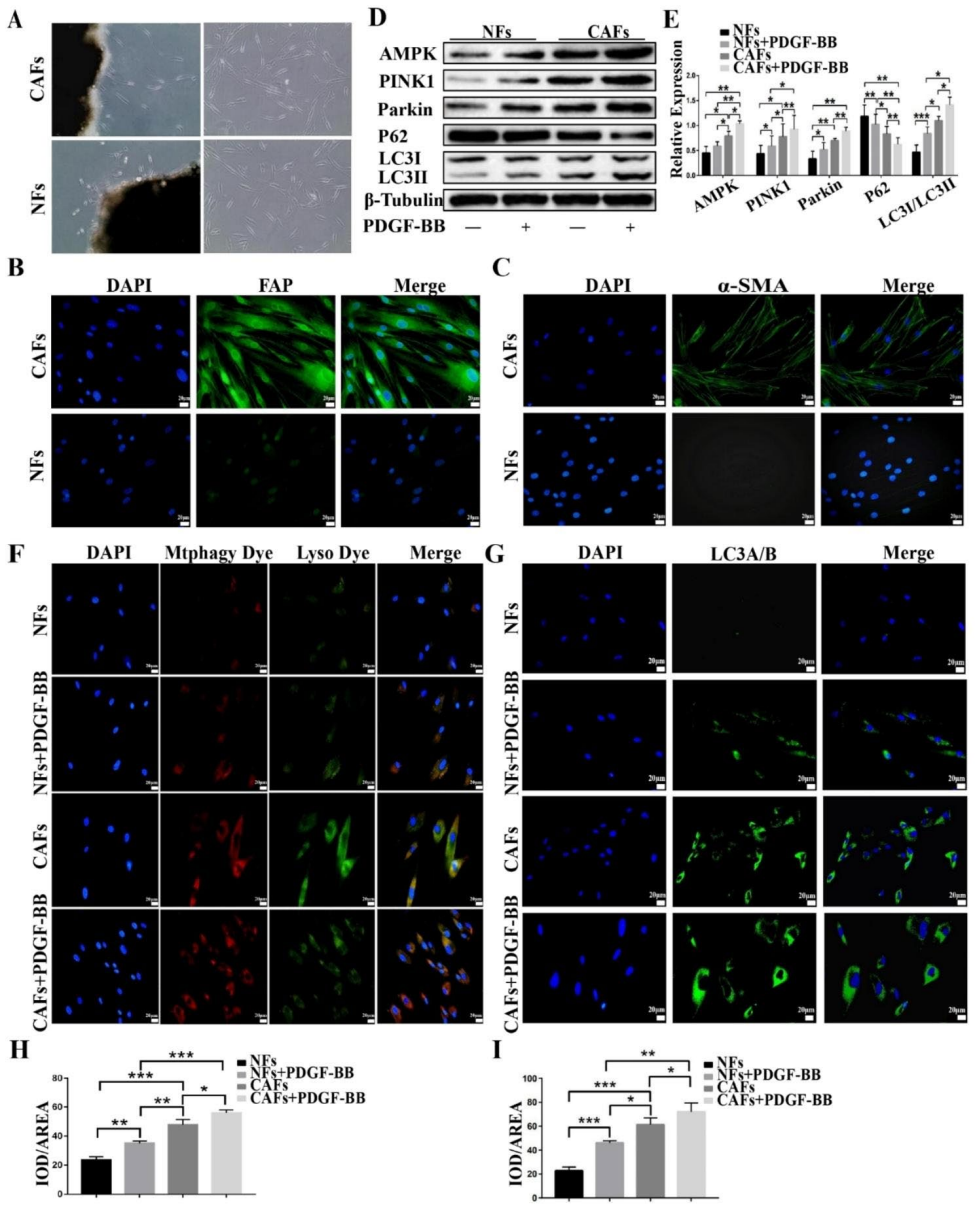
CAFs and NFs were isolated from tongue squamous cell carcinoma and adjacent tissues, respectively (**A**). Immunofluorescence staining revealed that both cell types were fibroblasts, with CAFs in cancer tissues showing high expression of α-SMA and FAP markers (magnification, 200×; scale bar, 20 μm) (**B**, **C**). Western blotting demonstrated that PDGF-BB treatment significantly enhanced the protein expression of LC3-II, AMPK, PINK1, and Parkin and decreased the expression of LC3-I and P62 in primary NFs and CAFs (**D**, **E**). Immunofluorescence staining showed that CAFs demonstrated higher levels of autophagy than NFs, and PDGF-BB treatment could enhance this effect (magnification, 200×; scale bar, 20 μm) (**F**–**I**)

CAFs are a crucial component of the tumor microenvironment and are known to play a significant role in tumor invasion, metastasis, and angiogenesis. To demonstrate the role of PDGF-BB in regulating autophagy and aerobic glycolysis, we obtained CAFs and NFs from tongue squamous cell carcinoma tissues and adjacent tissues (Fig. 5A). Immunofluorescence staining revealed that both cell types were fibroblasts derived from cancerous tissue and highly expressed CAF markers α-SMA and FAP (Fig. 5B, C). Subsequently, we used the same modeling approach to analyze the effect of PDGF-BB on autophagy in primary cells. The results showed that CAFs exhibited higher levels of autophagy (Fig. 5D–I) than NFs, and PDGF-BB further enhanced this effect.

### miR-26a-5p/ULK1 mediates PDGF-BB-induced reprogramming of glycolytic metabolism in activated fibroblasts

**Fig. 6:**
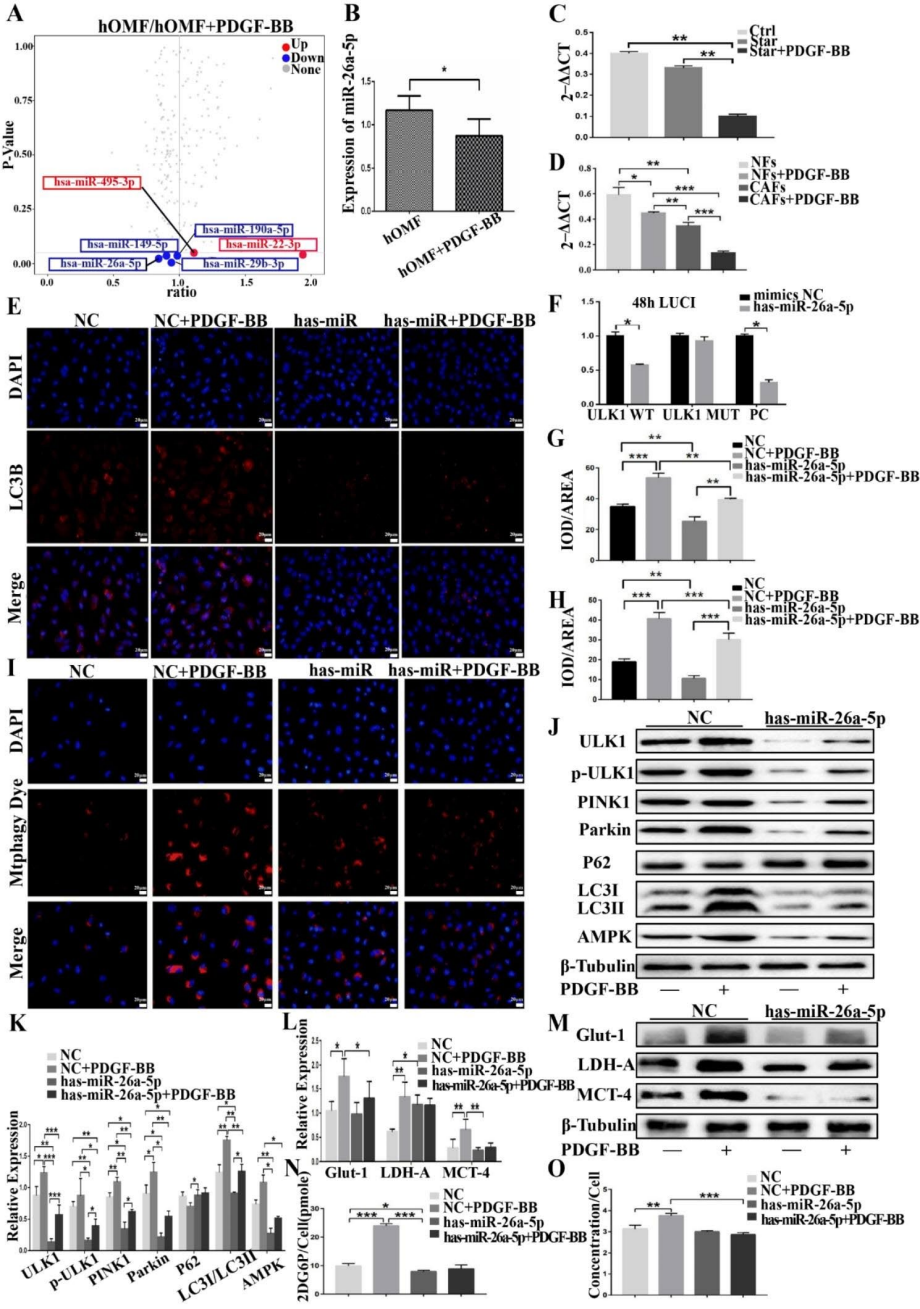
miRNA microarray analysis revealed a significant downregulation of miR-26a-5p expression (**A**). qRT-PCR indicated that PDGF-BB treatment downregulated miR-26a-5p expression in normal fibroblasts (**B**), primary cultured paraneoplastic (**C**), and cancer-associated fibroblasts (**D**). Dual luciferase reporter analysis confirmed ULK1 as its target gene (**F**). Immunofluorescence staining demonstrated that overexpression of miR-26a-5p inhibited PDGF-BB-induced intracellular LC3B expression (**E**, **G**) and mitochondrial autophagy (**H**, **I**) (magnification, 200×; scale bar, 20 μm). Western blotting indicated the downregulation of autophagy-related proteins ULK1, p-ULK1, Pink1, Parkin, P62, and LC3II (**J**, **K**). Glucose metabolism analysis demonstrated that miR-26a-5p inhibited PDGF-BB-induced glucose uptake (**N**) and lactate secretion (**O**) in hOMF cells, and Western blotting showed that miR-26a-5p inhibited PDGF-BB-induced expression of glucose metabolism-related proteins Glut-1, LDH-A, and MCT-4 (**L**-**M**)

To further investigate the mechanism by which PDGF-BB promotes the reprogramming of glucose metabolism in activated fibroblasts, we utilized miRNA chips and observed that the expression of miR-26a-5p was significantly downregulated (Fig. 6A). qRT-PCR further demonstrated that PDGF-BB downregulated the expression of miR-26a-5p in normal fibroblasts and primary cultured paracancerous and cancer-associated fibroblasts (Fig. 6B–D). We hypothesized that miR-26a-5p might play a significant regulatory role in PDGF-BB-induced autophagy. Subsequently, we revealed ULK1 to be the target gene of miR-26a-5p, as verified by the double luciferase analysis (Fig. 6F). We then overexpressed miR-26a-5p in hOMF cells and performed the previously described intervention methods to assess its impact on glucose metabolism and mitophagy. Immunofluorescence showed that overexpression of miR-26a-5p inhibited PDGF-BB-induced intracellular LC3B expression (Fig. 6E and G) and mitophagy (Fig. 6H–I). Western blotting indicated that the expression levels of autophagy-related proteins ULK1, p-ULK1, Pink1, Parkin, P62, and LC3II were significantly downregulated (Fig. 6J-K). Glucose metabolism analysis showed that miR-26a-5p inhibited PDGF-BB-induced glucose uptake and lactate secretion in star-treated hOMF cells (Fig. 6N, O). Western blotting further validated that miR-26a-5p inhibited the expression of PDGF-BB-induced glucose metabolism-related proteins Glut-1, LDH-A, and MCT-4 (Fig. 6L, M).

### PDGF-BB-induced CAFs promote the proliferation, invasion, Metastasis, and PDGF-BB secretion of Cal-27 under Starvation conditions

**Fig. 7:**
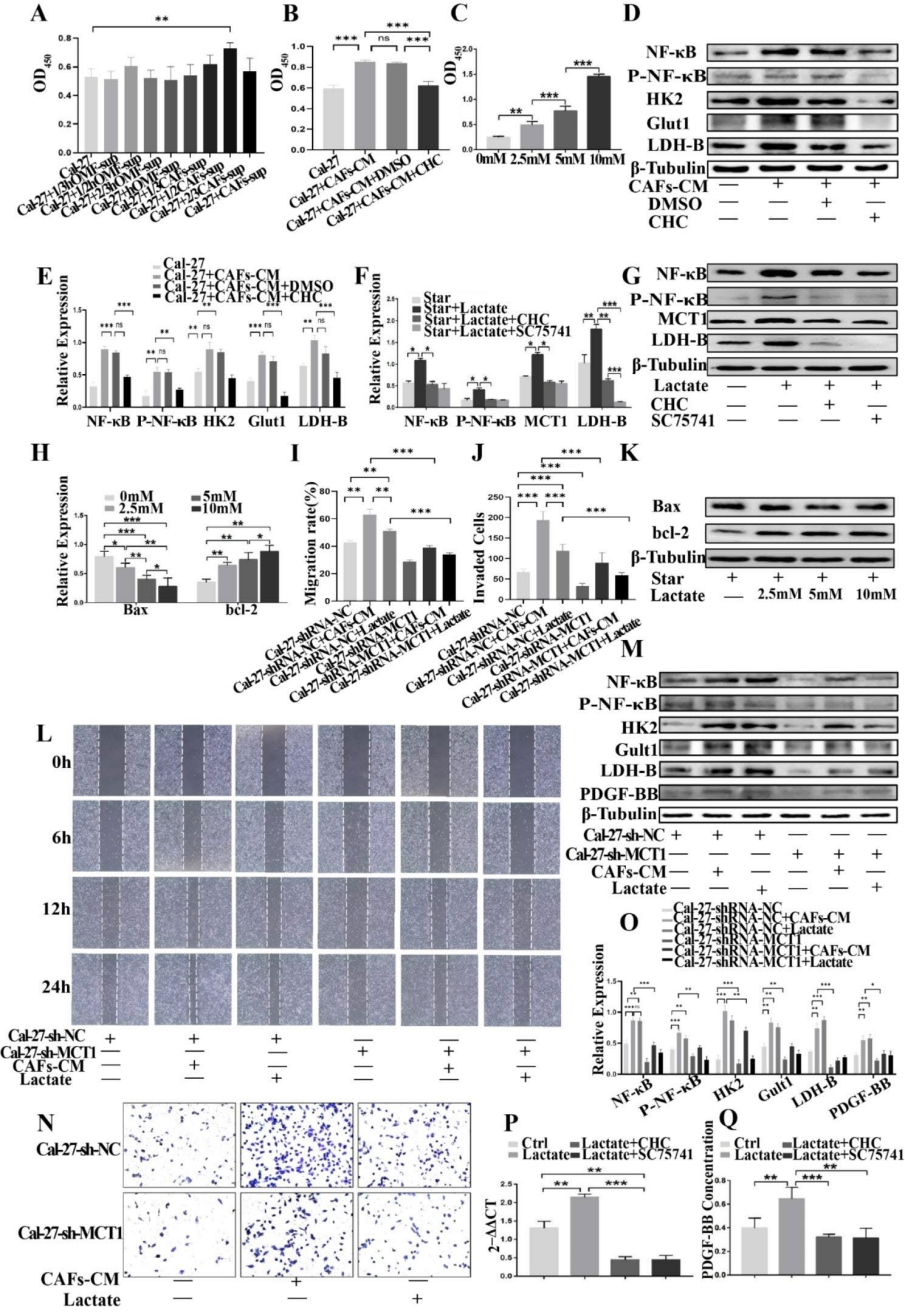
A 2/3 volume of CAF supernatant significantly promoted Cal-27 cell proliferation (**A**). CHC inhibited the proliferative effect of CAF supernatant (**B**). Lactic acid directly stimulated Cal-27 cells and promoted proliferation in a concentration-dependent manner (**C**). Western blotting that CAF supernatant upregulated the expression of NF-κB, p-NF-κB, HK2, Glut1, and LDHB proteins in Cal-27 cells (**D**, **E**), enhanced anti-apoptotic protein Bcl-2, and decreased pro-apoptotic protein Bax levels (**H**, **K**). CHC and SC75741 both inhibited the induction of NF-κB, p-NF-κB, MCT-1, LDH-B, HK2, and Glut1 protein (**F**, **G**) by lactate. qRT-PCR (**P**) and ELISA showed that lactate promoted the expression and secretion of PDGF-BB in Cal-27 cells, which was also inhibited by CHC and SC75741 (**Q**). After the knockdown of MCT1 gene expression in Cal-27 cells, CAF-derived lactate promoted diminished the migration (**I**, **L**) and invasion (**J**, **N**) abilities of tongue squamous carcinoma cells. In addition, the expression of NF-κB, p-NF-κB, PDGF-BB, HK2, Glut1, and LDHB proteins in Cal-27 cells was subsequently reduced (**M**, **O**)

It is well established that tumor cells in the tumor microenvironment can secrete a plethora of cytokines to induce mesenchymal cells to undergo metabolic reprogramming and provide the necessary nutrients for their survival. The present study has demonstrated that PDGF-BB can enhance CAF aerobic glycolysis and result in the secretion of a high level of lactic acid. The NF-κB signaling pathway is known to have significant regulatory effects on tumor growth and can promote PDGF-BB expression [23]. To further analyze the role of PDGF-BB, we indirectly co-cultured PDGF-BB-induced CAFs with the tongue squamous carcinoma cell line Cal27, and the results demonstrated that using a medium containing 2/3 PDGF-BB-induced CAF supernatant can significantly promote Cal27 proliferation (Fig. 7A), which could be reversed by CHC (MCT Inhibitor) (Fig. 7B). These results suggest that lactic acid plays a crucial role in this process. We directly stimulated Cal27 cells with varying concentrations of lactic acid and found that it promoted Cal27 cell proliferation in a concentration-dependent manner (Fig. 7C). Moreover, the expression levels of NF-κB, p-NF-κB, HK2, Glut1, LDHB, and anti-apoptotic protein Bcl-2 were upregulated (Fig. 7D, E), while the expression of pro-apoptotic protein Bax was decreased (Fig. 7H, K). To further elucidate the mechanism of action of lactic acid, we used CHC and SC75741 (NF-κB inhibitors). Western blotting showed that both CHC and SC75741 could inhibit the lactic acid-induced expression of NF-κB, p-NF-κB, MCT-1 (transport lactic acid into cells), LDH-B (convert lactic acid to pyruvate), HK2, and Glut1 (Fig. 7F, G). Surprisingly, qRT-PCR and ELISA demonstrated that lactic acid could promote Cal-27 cells to express and secrete PDGF-BB, while CHC and SC75741 inhibited this effect (Fig. 7P, Q). Furthermore, the knockdown of MCT1 gene expression in Cal-27 cells resulted in weakened migration (Fig. 7I, L) and invasion (Fig. 7J, N) abilities, and the protein expression levels of NF-κB, P-NF-κB, PDGF-BB, and the above key glycolytic enzymes were consequently reduced (Fig. 7M, O). These results indicate that lactate regulates Cal-27 glycolysis and PDGF-BB secretion through the activation of the NF-κB pathway by MCT1.

### PDGF-BB-induced CAFs promoted the growth of mixed xenograft tumors in Balb/c mice

**Fig. 8:**
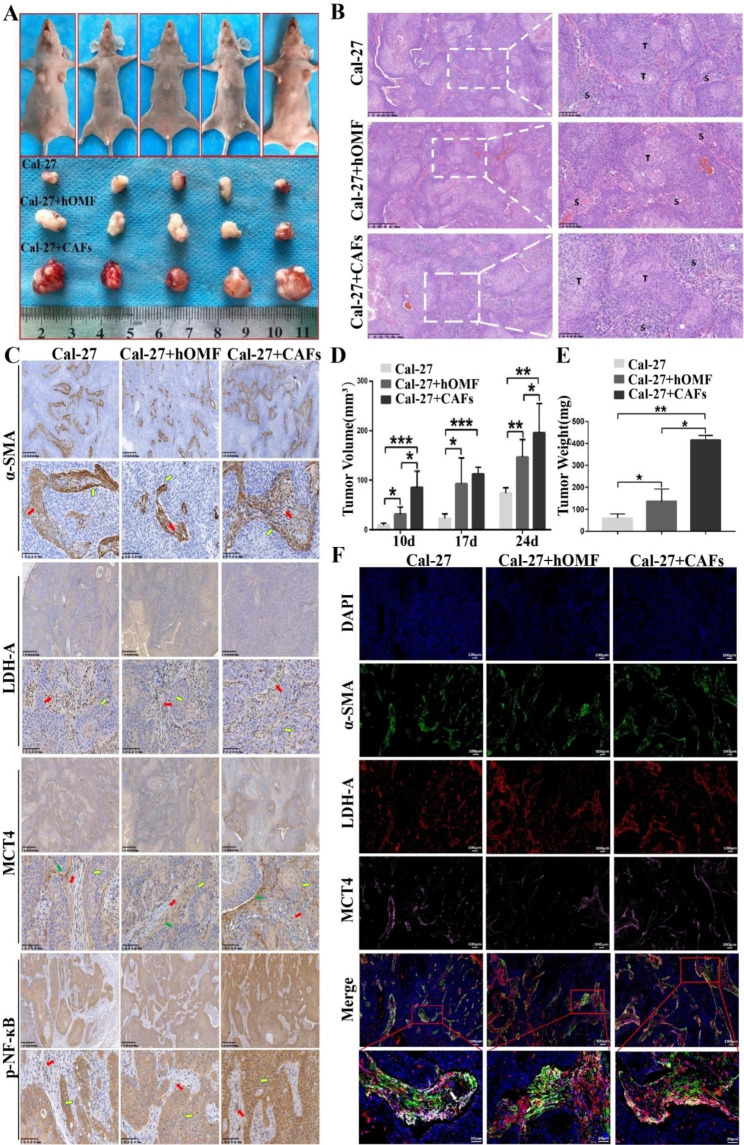
Mixed xenografts were assessed before and after tumor removal (**A**). HE staining revealed a large number of mesenchymal components visible in the tumor tissues of each group (scale bars 400 μm; 100 μm) (**B**). Immunohistochemical staining demonstrated that α-SMA, MCT-4, and LDH-A were predominantly expressed in the mesenchyme, while p-NF-ĸB was mainly expressed in tumor cells (scale bar 400 μm; 100 μm) (**C**). PDGF-BB-induced CAFs were found to promote tumor volume (**D**) and weight (**E**). Multiplex immunofluorescence co-localization staining revealed significant co-localization of α-SMA, MCT-4, and LDH-A in the mesenchyme, which was most pronounced adjacent to tumor cells (scale bar 100 μm; 20 μm) (**F**)

To investigate the effects of PDGF-BB on tumor development in vivo, we assessed tumor volume and weight in Balb/c mice challenged with mixed tumor xenografts. Our findings indicated that hOMFs and PDGF-BB-induced CAFs could promote tumor growth. However, the role of PDGF-BB-induced CAFs was more significant (Fig. 8A, D, and E). In addition, we performed a histopathological analysis of the tumor tissue. HE staining revealed the presence of a large number of interstitial components in the tumor tissue of all groups (Fig. 8B). Immunohistochemical staining demonstrated that α-SMA, MCT-4, and LDH-A were predominantly expressed in the stroma, while p-NF-ĸB was expressed in tumor cells (Fig. 8C). Given that the interstitial cells contain numerous cellular components, we conducted multiple immunofluorescence colocalization stainings for α-SMA, MCT-4, and LDH-A to better understand the glucose metabolism of CAFs in the tumor microenvironment. The three markers exhibited obvious colocalization in the stroma, particularly in close proximity to tumor cells (Fig. 8F).

Importantly, the cotransplantation of Cal-27 cells with MCT1 gene expression knocked down, and CAFs resulted in weakened proliferation-promoting effects of CAFs and reduced tumor mass and volume (Figure S1A–D). To confirm the effect of CAF-derived lactic acid on tumor cell metabolic reprogramming, we performed multiple immunofluorescence colocalization stainings for MCT1, Glut1, and LDH-B. The three markers exhibited clear colocalization in the cytoplasm (Figure S1E).

## Discussion

Cancer-associated fibroblasts (CAFs) are a major component of the tumor stroma and contribute to tumor invasion and metastasis in the complex network system of the tumor microenvironment [25]. Similar to normal fibroblasts, CAFs express vimentin but not keratin. CAFs can arise from normal fibroblasts that are directly activated by cytokines secreted by tumor cells, such as TGF-β1 and PDGF-BB, and express specific markers, including α-SMA, FAP, and PDGFR-β [26]. In this study, we observed significant α-SMA and PDGFR-β expression in the interstitial area of cancer tissues but not in adjacent tissues. PDGF-BB was mainly expressed in tumor cells. CAFs and adjacent cancer fibroblasts were isolated from tongue squamous cell carcinoma and adjacent tissues, and both expressed vimentin but not keratin, while CAFs highly expressed α-SMA and FAP. To further analyze the relationship between PDGF-BB and the formation of CAFs in the tumor microenvironment, we employed a starved hOMF cell model to simulate the nutritional deprivation of the tumor microenvironment in vitro. Our results showed that starvation can activate fibroblasts, consistent with Bernard M’s findings, and PDGF-BB can enhance this activation state, resulting in increased α-SMA and FAP expression [27].

Nutritional deficiency and energy limitation are significant characteristics of the tumor microenvironment. Studies have shown that nutritional deficiency can inhibit tumor proliferation by reducing aging-related inflammatory responses, increasing glucocorticoids, and reducing angiogenesis [28]. In response to this challenge, tumor cells not only change their energy metabolism from aerobic phosphorylation to aerobic glycolysis (the Warburg effect) [29] but also educate and induce stromal cells, especially CAFs, to reprogram and produce high-energy metabolites, such as lactic acid and/or ketones, which provide energy substances for tumor proliferation and metastasis (the reverse Warburg effect) [30, 31]. Our research revealed that MCT-4 and MCT-1 are highly expressed in oral squamous cell carcinoma tissue. MCT-4 is expressed in tumor interstitial fibroblasts and tumor cells, while MCT-1 is mainly expressed in the frontal region of growth in contact with the mesenchyme. Subsequent in vitro studies confirmed that PDGF-BB upregulated the aerobic glycolysis indicators GLUT-1, LDH-A, and MCT-4, which effluxes lactic acid, in star-treated hOMF cells and primary cultured CAFs. The lactic acid content in the culture supernatant increased significantly. In the tumor microenvironment, cancer cells can transport CAF-derived lactic acid into their intracellular space via MCT-1. This lactic acid is then converted into pyruvate by LDHB, which enters the mitochondrial TCA cycle and acts as a signaling molecule [17]. To further study the metabolic interaction between these cells, we used PDGF-BB-activated fibroblast supernatant and lactic acid to stimulate Cal-27 cells. The results showed enhanced proliferation ability, increased expression of monocarboxylic acid transporter-1, and secretion of PDGF-BB. Meanwhile, the NF-κB signaling pathway was significantly activated. Subsequently, knockdown of the MCT1 gene in Cal-27 cells resulted in a significant decrease in the pro-proliferative, invasive, and migratory abilities of PDGF-BB-activated fibroblast supernatant and lactate and the secretion of PDGF-BB.

The cotransplantation model of subcutaneous cells in nude mice revealed that PDGF-BB-activated fibroblasts have significant tumorigenic effects. Tumor interstitial fibroblasts showed colocalization of α-SMA, LDH-A, and MCT-4, while p-NF-κB was mainly expressed in tumor cells. Knockdown of MCT1 in Cal-27 cells reduced the pro-proliferative effect of PDGF-BB-activated fibroblasts. These findings suggest that PDGF-BB induces the reprogramming of glucose metabolism in activated fibroblasts, leading to the production of lactic acid that provides nutrients for the rapid proliferation of tumors. This, in turn, further promotes PDGF-BB secretion by tumor cells through the NF-κB signaling pathway, forming a positive interaction loop that supports the rapid proliferation of tumors [23].

Autophagy is a cellular catabolic mechanism that facilitates the recycling of organelles, lipids, and proteins, playing a crucial role in maintaining cellular homeostasis and providing a source of energy [32]. Sanchez et al. reported that stromal cells in the nutrient-deprived core utilized autophagy to sustain the surrounding cells [33]. According to Liu et al., CAFs experience energy deprivation in the tumor microenvironment. CAFs activate mitochondrial autophagy to meet the energy demands of nearby cancer cells [34]. Consistent with previous findings, the MDC results demonstrated that PDGF-BB enhanced Star-induced autophagy. The upregulation of mitophagy-related proteins Pink1, Parkin, and conversion of LC3 I to II, coupled with the downregulation of P62, indicate that PDGF-BB enhances Star-induced mitophagy. Our TEM and fluorescence imaging data further supported the notion that PDGF-BB promotes mitophagy. Mitochondrial autophagy causes the blockage of oxidative phosphorylation, thereby enhancing aerobic glycolysis and resulting in the secretion of large levels of lactic acid, which is a significant characteristic of tumor microenvironmental stress [13]. Our research also revealed that PDGF-BB increased aerobic glycolysis levels and lactic acid secretion, which could be prevented by autophagy inhibitors, such as mdivi-1.

Substantial research has focused on the impact of miRNAs on mitochondrial function in recent years. Chen et al. [35] found that miR-210 downregulated mitochondrial iron sulfur cluster scaffold protein (ISCU) and cytochrome C oxidase assembly protein-10 (COX10), regulated the mitochondrial electron transport chain, and enhanced glycolytic metabolism. Puisségur et al. [36] confirmed that miR-210 could induce the loss of mitochondrial membrane potential and abnormal mitochondrial phenotypes and also targeted the electron transport chain (ETC), a key regulator of mitochondrial function, affecting cell metabolism and apoptosis. Our research revealed that PDGF-BB significantly reduced the expression of miR-26a-5p during fibroblast activation. Furthermore, miR-26a-5p was downregulated in CAFs derived from cancer tissues, indicating that miR-26a-5p played a critical role in regulating fibroblast activation. Target gene prediction suggested that the autophagy-related gene ULK1 was a target of miR-26a-5p [37]. Upon the overexpression of miR-26a-5p in hOMF cells, PDGF-BB-induced mitochondrial autophagy and aerobic glycolysis were significantly inhibited. Related studies have also shown that the downregulation of miR-26a/b in hepatoma cells enhances ULK1 expression, promoting autophagy and chemotherapy resistance [38].

**Fig. 9:**
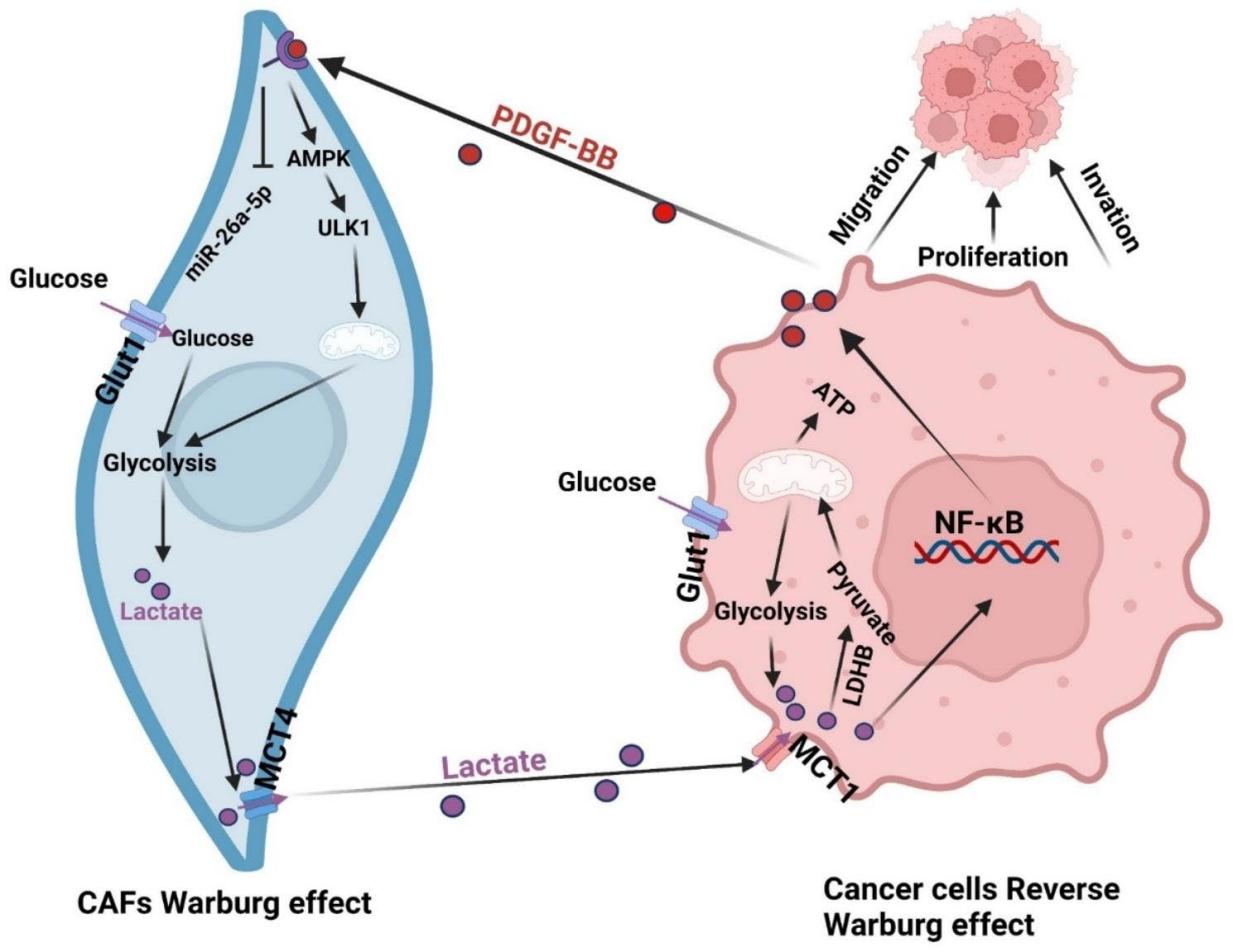
Metabolic symbiosis model diagram

In summary, our comprehensive analysis underscores the pivotal role of PDGF-BB in instigating the biogenesis of CAFs and orchestrating a metabolic shift, culminating in amplified tumor progression, as evidenced in Fig. 9. Intriguingly, we delineated a mechanistic pathway wherein autophagy-triggered mitochondrial aberrations within stromal fibroblasts bolster lactate synthesis, consequently diverting cellular metabolic machinery toward glycolysis. In addition, the autophagic response elicited by PDGF-BB in fibroblasts augments neoplastic cell proliferation, primarily through providing lactate, a potent mitochondrial substrate that neighboring malignancies harness via oxidative phosphorylation. Notably, this intricate interplay is thwarted by the autophagy antagonist mdivi-1 and miR-26a-5p. The preponderance of evidence from our investigations intimates the instrumental contribution of PDGF-BB emanating from oncogenic cells in propelling tumorigenicity by invoking a catabolic metabolic trajectory in stromal compartments. Collectively, this research elucidates the nuanced role of PDGF-BB in the pathogenesis of oral tongue squamous cell carcinoma, spotlighting its propensity to sculpt a conducive oncogenic microenvironment via stromal metabolic modulation.

### Electronic Supplementary Material

Below is the link to the electronic supplementary material.

Supplementary Material 1

## Data Availability

The datasets generated and/or analyzed during the current study are available from the corresponding author on reasonable request.
