# Supplementary material for: PDGF-BB accelerates TSCC via fibroblast lactates limiting miR-26a-5p and boosting mitophagy
Source: Cancer Cell Int. 2024 Jan 2;24:5. doi: 10.1186/s12935-023-03172-6 (PMC10763357; doi:10.1186/s12935-023-03172-6)
Supplement: Supplementary file 1 — Supplementary Material 1 [file 12935_2023_3172_MOESM1_ESM.docx]

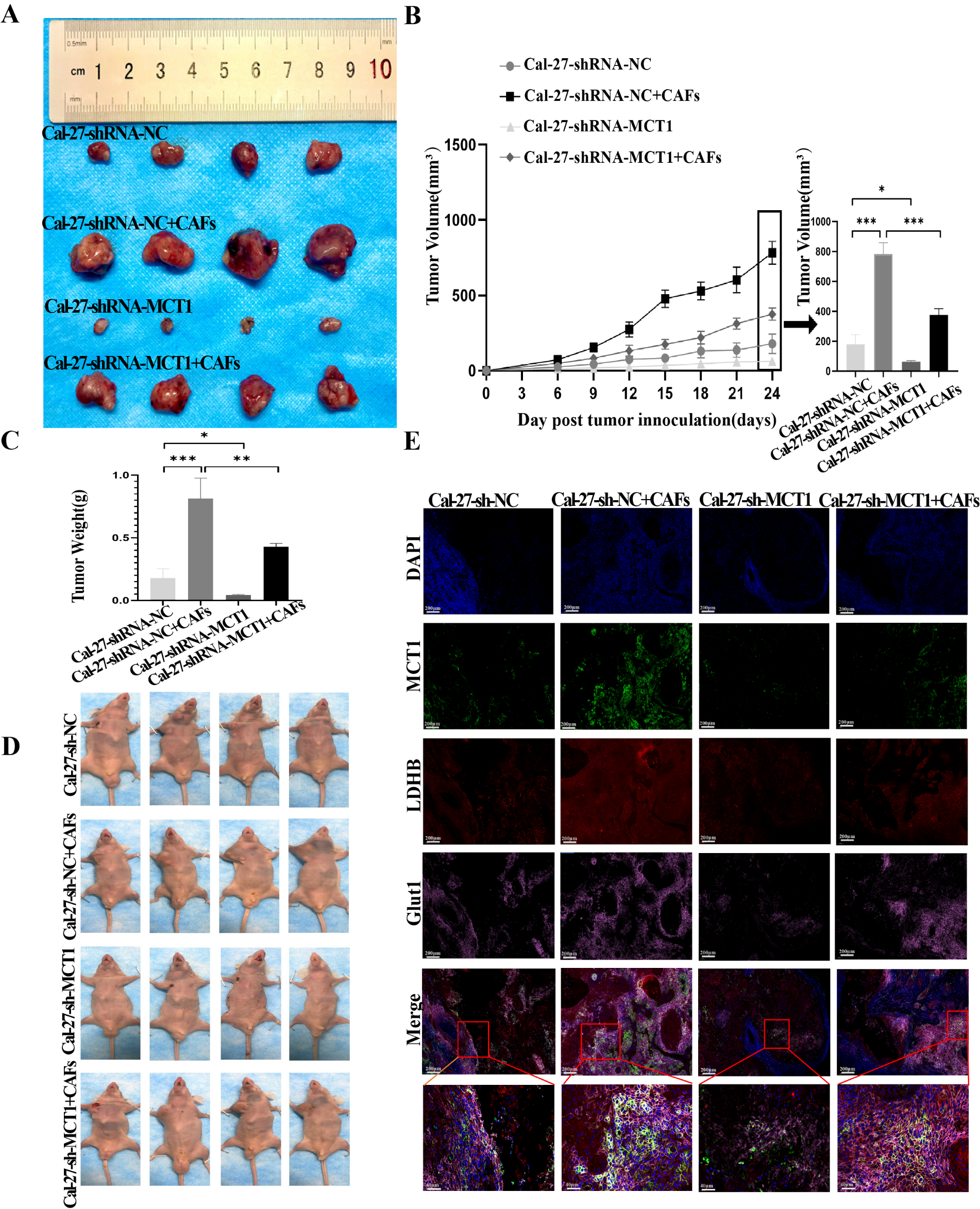


Fig.S1

After the extraction of mixed xenograft tumors, knockdown of MCT1 gene expression was performed in Cal-27 cells (A). This resulted in a reduction in the pro-proliferative effect of CAFs and a decrease in the tumor mass (B) and volume (C). Before extracting mixed xenograft tumors(D). Multiplex immunofluorescence co-localization staining revealed significant co-localization of MCT1, Glut1, and LDH-B (scale bar 200 µm; 40 µm) (E).
